# Evaluation of a novel contrast‐enhanced fluoroscopy protocol for endoscopic retrograde cholangiopancreatography in a phantom model (with video)

**DOI:** 10.1002/deo2.70087

**Published:** 2025-02-23

**Authors:** Takaoki Hayakawa, Masayasu Horibe, Eisuke Iwasaki, Fateh Bazerbachi, Yuki Suno, Tsubasa Sato, Haruka Okada, Yuki Nakajima, Yosuke Mizukami, Atsuto Kayashima, Takashi Seino, Shintaro Kawasaki, Takanori Kanai

**Affiliations:** ^1^ Department of Internal Medicine Division of Gastroenterology and Hepatology School of Medicine, Keio University Tokyo Japan; ^2^ CentraCare, Interventional Endoscopy Program, St. Cloud Hospital St. Cloud USA; ^3^ Division of Gastroenterology Hepatology and Nutrition University of Minnesota Minneapolis USA; ^4^ Office of Radiation Technology Keio University School of Medicine Tokyo Japan; ^5^ Center for Diagnostic and Therapeutic Endoscopy, School of Medicine, Keio University Tokyo Japan

**Keywords:** cholangiopancreatography, fluoroscopy, phantoms, radiography, radiologic phantom

## Abstract

**Objectives:**

This study evaluated a novel contrast‐enhanced (CE) fluoroscopy protocol for endoscopic retrograde cholangiopancreatography, which optimizes image processing to enhance contrast of devices and contrast media. We compared the CE protocol with the conventional standard protocol to assess its potential for reducing radiation exposure while improving image visibility.

**Methods:**

The study utilized a multidirectional fluoroscopy unit and phantoms to evaluate the new CE protocol against the conventional protocol. Comparisons included radiation dose rates, spatial resolution, and concentration resolution under various fluoroscopic conditions. The investigation aimed to determine if the CE protocol offered improved visibility while potentially reducing radiation exposure.

**Results:**

Three CE protocol modes (LOW‐7.5 fps, MID‐3.75 fps, and LOW‐3.75 fps) achieved lower dose rates than the standard MID‐7.5 fps mode commonly used in clinical practice. Dynamic spatial resolution was significantly superior in all three CE modes compared to the standard protocol (*p* < 0.0167). Static spatial resolution did not differ significantly between protocols. Only the CE MID‐3.75 fps mode showed superior concentration resolution compared to the standard protocol (*p* < 0.00833).

**Conclusions:**

The novel CE fluoroscopy protocol provides superior dynamic spatial resolution in endoscopic retrograde cholangiopancreatopgraphy while reducing radiation exposure, potentially enhancing procedure guidance and safety for both patients and clinicians.

## INTRODUCTION

Endoscopic retrograde cholangiopancreatography (ERCP) relies on fluoroscopy for instrument guidance and duct visualization. Due to its complexity and extended duration, ERCP typically involves higher radiation exposure compared to other fluoroscopy‐assisted endoscopic procedures.^1^ Abe et al. reported that ERCP, especially in its therapeutic applications, resulted in higher radiation doses (air kerma: 93 mGy; kerma‐area product: 26 Gy·cm^2^) and longer fluoroscopy times (14 min) than other gastrointestinal fluoroscopic procedures.^2^ Therefore, minimizing radiation exposure during ERCP is crucial for the safety of patients, endoscopists, and staff.

While various methods such as pulsed fluoroscopy, protective clothing, and under‐couch type C‐arms have been employed to reduce radiation dose, studies on optimized fluoroscopy protocols are limited.^3^, ^4^, ^5^, ^6^

In 2023, a novel contrast‐enhanced (CE) protocol was developed for the Ultimax‐i system (Canon Medical Systems Corporation), featuring a multidirectional C‐arm with a 42 × 42 cm flat panel detector. This protocol aims to enhance image quality and device visibility while reducing radiation exposure compared to conventional systems. Through optimized contrast processing for devices and contrast media, the CE protocol maintains visibility at lower radiation doses and can be readily activated via the control panel.

The present study sought to evaluate whether the CE protocol, operating at low radiation doses, could provide superior visibility compared to the conventional fluoroscopy protocol (standard protocol), specifically in terms of spatial resolution and concentration resolution.

## METHODS

### Equipment

This study was conducted at Keio University Hospital in Japan using the Ultimax‐i system. The system's X‐ray conditions were automatically set using Auto Brightness Control (ABC), mirroring clinical settings, with an additional 0.2 mm Cu filter. Three fluoroscopic modes were employed: low‐dose (LOW), intermediate‐dose (MID), and normal‐dose (NORMAL), each supporting pulse rates of 1, 2.14, 3.75, 7.5, and 15 fps.

The Ultimax‐i supports both the novel CE protocol and the conventional fluoroscopic protocol. The CE protocol enhances image quality through optimized image processing and ABC (luminance adjustment), providing superior contrast for devices and contrast media compared to the conventional mode. By tailoring image display parameters to the device or contrast agent and optimizing a combination of (1) gradation processing, (2) dynamic range compression, and (3) frequency enhancement, the protocol enhances the visibility of low‐contrast areas while maintaining overall image contrast. Gradation processing adjusts the gradation levels of the image to make the target area easier to observe. Dynamic range compression adjusts brightness to allow observation of a wide range of gradation levels simultaneously. Frequency enhancement emphasizes the characteristics of devices and contrast agents by analyzing and enhancing specific frequencies. These approaches allow for improved visualization of low‐contrast areas while maintaining overall image contrast. Furthermore, the CE protocol optimizes display luminance and ABC specifically for image processing, resulting in enhanced visibility even at lower radiation doses.

### Measurement and evaluation methods

#### Evaluation process

Our evaluation followed a systematic approach. First, we compared dose rates between the standard protocol and the new CE protocol. Based on previous studies,^7^, ^8^ we defined MID‐7.5 fps as our standard mode. We then identified CE modes that demonstrated lower dose rates than this standard mode. These identified CE modes were subsequently compared with the standard mode across three parameters: dynamic spatial resolution, static spatial resolution, and concentration resolution.

#### Dose measurements

**FIGURE 1:**
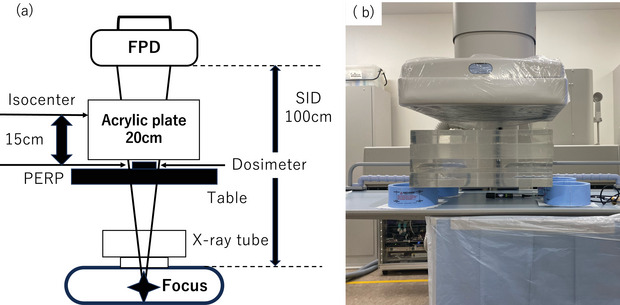
Geometrical setup for dosimetry measurement. Schematic (a) and photographic (b) representation of the setup for dosimetry at the patient entrance reference point (PERP), FPD: flat panel detector; SID, source to image receptor (FPD) distance.

Radiation dosage was measured using an AGDM+ dosimeter (Toyo Medic Co. Ltd). The experimental setup featured a field‐of‐view size of 25 × 25 cm (M2 mode), with a 20 cm thick acrylic plate (50 × 50 cm) serving as the scatterer. The geometrical arrangement of the Flat Panel Detector (FPD) was as follows: the dose rate per minute (mGy/min) was measured at the patient entrance reference point (PERP), situated 15 cm from the C‐arm rotation center (isocenter) to the X‐ray tube, as illustrated in Figure 1.^9^, ^10^

Measurements were conducted for both standard and CE protocols, using LOW and MID modes. While pulse rates of 1, 2.14, 3.75, 7.5, and 15 fps are available, we restricted our evaluation to rates of 3.75 fps and above, as lower rates produce excessively discontinuous images that make device manipulation impractical in clinical settings. Each condition was assessed three times for 30 seconds, with the average taken as the final value. The collected images had a resolution of 1024 × 1024 pixels.

Results were expressed as mean and standard deviation (SD). These measurements were used to identify CE modes with lower doses than the standard MID‐7.5 fps mode, which is commonly used in clinical practice.

#### Visual evaluation of dynamic spatial resolution

**FIGURE 2:**
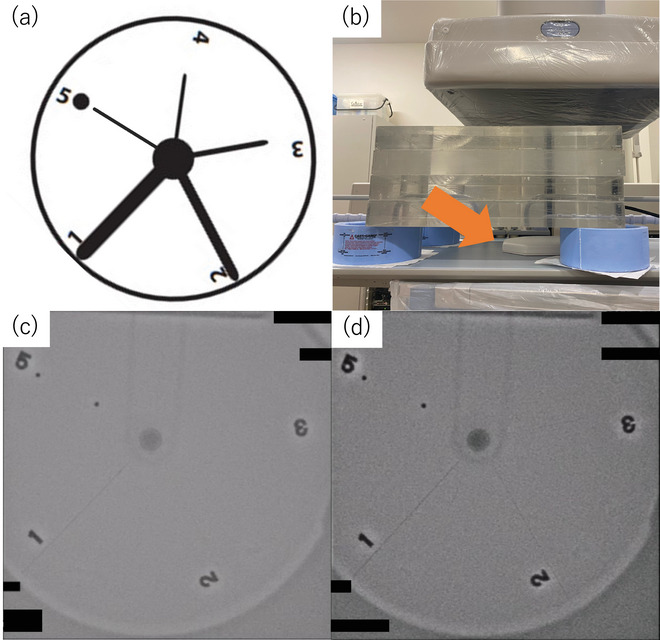
Evaluation of dynamic spatial resolution. A rotating wire phantom (NEMA SCA&I Rotating Spoke 901–13, Virginia, USA) was used to assess dynamic spatial resolution based on the visibility of thin wires. (a) Diagram of the phantom (b) Placement of the phantom (arrow) at the patient entrance reference point. (c) Fluoroscopic image using the standard protocol at MID‐7.5 fps. (d) Fluoroscopic image using contrast‐enhanced protocol at LOW‐7.5 fps.

Dynamic spatial resolution was assessed using a cardiac rotating wire phantom (NEMA SCA&I Rotating Spoke 901–13), as no ERCP‐specific phantom currently exists. The setup consisted of‐ Base: acrylic plate (30 × 30 × 20 cm), Rotation: 30 rpm (110 V/60 Hz), motor‐driven via a belt connection, Test elements: Five stainless steel wires (0.022, 0.016, 0.012, 0.009, and 0.005 inches) arranged at 72° intervals rotating wire phantom (NEMA SCA&I Rotating Spoke 901–13). As a rotating phantom specifically designed for ERCP does not currently exist, we utilized the cardiac phantom as a substitute for this study. Figure 2 illustrates the rotating wire phantom and its geometric arrangement.

Fluoroscopic recordings were made for ten seconds using both the standard and CE protocols, at pulse rates of 3.75 fps and 7.5 fps, and at LOW and MID doses. The evaluation method involved determining the number of the thinnest wires that could be recognized (APPENDIX S1).

Video images were evaluated independently and randomly. All evaluators were blinded to the protocol type (Standard or CE), frame rate, and dose level (LOW or MID) during the assessment. The evaluation order was randomized for each evaluator. If variation was observed in the evaluation results, the average score of all evaluators was adopted as the final value. Assessment conditions mimicked real‐world clinical settings, with images displayed on a quad screen of a 55‐inch monitor (MDSC‐8255; Barco), simulating the split‐screen view typically used in ERCP procedures. The evaluation panel included six endoscopists and five certified radiology technologists, each with at least seven years of experience in ERCP and fluoroscopy, respectively. All evaluators provided consent for the publication of their assessment results in this study.

#### Visual evaluation of static spatial and concentration resolution

**FIGURE 3:**
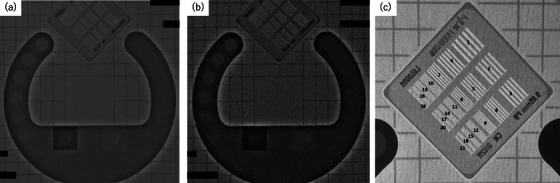
Evaluation of static spatial and concentration resolution. Test object (X‐Check FLU L981319; PTW‐Freiburg) used for assessing fluoroscopic and indirect radiographic X‐ray equipment. (a) Fluoroscopic image using the standard protocol at MID‐7.5 fps. (b) Fluoroscopic image using contrast‐enhanced protocol at LOW‐7.5 fps. (c) Fluoroscopic image showing 21 slits of varying thickness used to evaluate static spatial resolution based on the maximum number of recognizable slits.

Static spatial resolution and concentration resolution were evaluated using a test object designed for constancy tests of fluoroscopic and indirect radiographic X‐ray equipment (X‐Check FLU L981319; PTW‐Freiburg; Figure 3). This phantom featured 21 slits of varying thickness and 19 circular contrasts of 10‐mm diameter (0.5% ‐ 20.0%), representing the linear grayscale value of the image on an aluminum disk at 70 kV. The phantom was positioned on a 20‐cm acrylic plate within the fluoroscopy apparatus. Fluoroscopic recordings, each lasting ten seconds, were made using both the standard and CE protocols. Slits were assigned a number from 1 to 21 for the corresponding position, starting with the thickest slit. Static spatial resolution was quantified as “scored spatial resolution,” the number of the thinnest slit recognizable in the video recorded at each mode, and used for evaluation. Concentration resolution was assessed by counting the number of visible aluminum disks that could be clearly identified as circles. The evaluation process, conducted by the same 11 experts who assessed the dynamic spatial resolution, followed an identical methodology to that used in the dynamic spatial resolution evaluation.’’

#### Statistical analyses

Dose measurements were expressed as mean and SD. Visual assessments of dynamic spatial resolution, static spatial resolution, and concentration resolution were based on the number of visible wires, spatial resolution score, and number of visible aluminum disks, respectively. The Wilcoxon signed‐rank test was used for statistical analysis, and the Bonferroni correction was used for multiple comparisons. Specifically, the latter method was used to obtain the adjusted significance level (α'), which was then used to judge the probability value of the result of a significance test for each comparison pair. To assess the dynamic spatial resolution, α' was set at 0.017, which was calculated by dividing 0.05 by the number of tests (three). Similarly, the static spatial resolution and contrast resolution were assessed by setting α' to 0.0083, which was calculated by dividing 0.05 by the number of tests (six). Moreover, since each evaluation was conducted only once and the sample size was very small, the agreement of evaluation results among multiple evaluators was not assessed. All statistical analyses were performed using R Statistical Software (version 4.2.2; R Foundation for Statistical Computing).

## RESULTS

### Dose measurement

Table 1 shows the dose rates for each mode in the standard and new CE protocol. The dose rates of all CE modes were higher than those of the standard protocol under the same conditions. The overall dose rate in CE was 84.9% higher than in the standard protocol, and the dose rate of CE was 83.5% higher than that of the standard protocol at MID‐7.5 fps.

**TABLE 1:** Dose rate comparison. Comparison of dose rates between the standard protocol and the new contrast‐enhanced protocol across various modes.

Standard protocol (mGy/min)	Pulse rate		MID	LOW
	15 fps	Mean	11.08	5.68
		SD	0.37	0.07
	7.5 fps	Mean	5.42	2.87
		SD	0.13	0.07
	3.75 fps	Mean	2.63	1.39
		SD	0.06	0.04

Contrast‐enhanced (CE) protocol (mGy/min)	Pulse rate		MID	LOW
	15 fps	Mean	20.59	10.64
		SD	0.66	0.16
	7.5 fps	Mean	9.95	5.27
		SD	0.19	0.14
	3.75 fps	Mean	4.76	2.55
		SD	0.08	0.08

Only three CE modes demonstrated lower doses than the standard MID‐7.5 fps mode (commonly used in clinical practice): (1) LOW‐7.5 fps, (2) MID‐3.75 fps, and (3) LOW‐3.75 fps.

### Visual evaluation of dynamic spatial resolution

**FIGURE 4:**
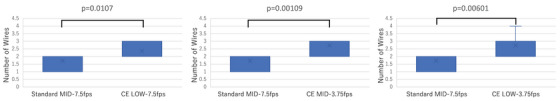
Box and whisker plots of comparison of dynamic spatial resolution. Visual assessment results comparing three contrast‐enhanced (CE) protocol conditions (LOW‐7.5 fps, MID‐3.75 fps, and LOW‐3.75 fps) with the standard protocol (MID‐7.5 fps). Note: Fractional values in the box plots represent statistical quartiles derived from the evaluators' integer scores.

Three CE modes (LOW‐7.5 fps, MID‐3.75 fps, and LOW‐3.75 fps) were compared to the standard MID‐7.5 fps mode, commonly used in clinical practice. Figure 4 shows the results of the visual evaluation of dynamic spatial resolution. Statistical analysis using the Wilcoxon signed‐rank test revealed that wire visibility was significantly superior in all three CE modes compared to the conventional mode (*p* < 0.0167 for each comparison). No statistically significant differences were found between endoscopist and radiology technologist evaluations after Bonferroni correction (all *p* > 0.0125).

### Visual evaluation of static spatial and concentration resolution

**FIGURE 5:**
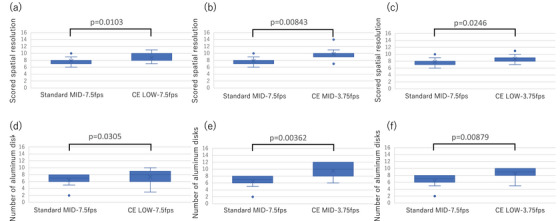
Box and whisker plots of static spatial and concentration resolution comparison. Visual evaluation results of: (a–c) static spatial resolution and (d–f) concentration resolution.

Figure 5 compares the static spatial resolution and concentration resolution between the CE protocol and the standard protocol. Static spatial resolution under any of the CE protocols did not differ significantly from that of the standard protocol. On the other hand, only CE MID‐3.75 fps had a concentration resolution superior to that of the standard mode (*p* < 0.00833). No statistically significant differences were found between endoscopist and radiology technologist evaluations after Bonferroni correction (all *p* > 0.0125).

## DISCUSSION

Our study demonstrates a nuanced relationship between radiation exposure and image quality in the CE protocol. While the CE protocol demonstrated higher radiation exposure compared to the standard protocol under the identical settings, three specific CE modes—LOW‐7.5 fps, MID‐3.75 fps, and LOW‐3.75 fps—achieved superior dynamic spatial resolution while operating at lower radiation doses than the commonly used standard protocol, MID‐7.5 fps.

Dynamic spatial resolution is an index of the clarity of X‐ray images of moving objects and is highly useful in real‐time procedural interventions.^11^ Static spatial resolution is an index of the degree to which fine structures can be distinguished in static images, whereas concentration resolution measures the ability to distinguish substances with differing concentrations.^11^ While objective metrics such as the contrast‐to‐noise ratio exist and are commonly used to evaluate these aspects;^11^ they were not suitable for evaluating the CE protocol. This is because the CE protocol is specifically designed to enhance contrast and can be affected by motion and timing artifacts. Therefore, we conducted blinded evaluations by experienced evaluators to assess image quality as perceived in clinical practice. The phantom studies revealed important technical capabilities of the CE protocol. Using a cardiac rotating phantom—chosen in the absence of ERCP‐specific testing equipment—we evaluated the visualization of guidewires ranging from 0.022 to 0.005 inches in diameter. Notably, two of our 11 evaluators successfully visualized the 0.009‐inch guidewire, demonstrating resolution capabilities beyond the typical 0.018‐inch minimum used in ERCP procedures. This enhanced visualization capability suggests potential benefits for complex procedures requiring precise device manipulation.

Our findings showed that the CE mode had superior dynamic spatial resolution under all three conditions: LOW‐7.5 fps, MID‐3.75 fps, and LOW‐3.75 fps. This suggests that the CE mode can facilitate the observation of device movements under real‐world clinical conditions. However, these three conditions did not significantly differ from those of the conventional mode in terms of static spatial resolution, with only MID‐3.75 fps in CE showing superior spatial resolution. These results demonstrate that the CE protocol provides superior dynamic spatial resolution at lower radiation doses compared to the conventional protocol. This improvement is likely attributable to the advanced image processing capabilities of the CE mode. While the CE protocol did not show significant improvements in static spatial resolution and contrast resolution across all modes, it maintained performance comparable to the conventional protocol. This suggests that the CE protocol does not compromise these aspects of image quality while offering benefits in other areas. Although increasing the sample size might reveal subtle differences, the current study found no statistically significant distinctions. It remains unclear which of the three identified CE modes is the most optimal. Nevertheless, we recommend initiating procedures with the lowest‐radiation CE LOW‐3.75 fps and increasing the radiation dose if device visibility proves inadequate. The CE protocol is its user‐friendly interface, which allows for seamless transitions between modes via the control panel.

The CE protocol may be particularly valuable for complex ERCP procedures requiring fine guidewire manipulation. For example, it may be applied to good effect in examinations such as endoscopic transpapillary gallbladder drainage (ETGBD). A retrospective study at our center found that using LOW‐3.75 fps in CE instead of the conventional mode could potentially reduce the total dose from 124.2 to 58.3 mGy during a single ETGBD examination, given an average exposure time of 22.9 min (Data not shown). The CE protocol may help reduce radiation exposure time and dose in complex procedures by providing clearer images. However, these findings should be considered preliminary, as clinical practice often combines radiography with fluoroscopy. Prospective clinical studies are needed to validate these results.

These findings gain particular relevance against the backdrop of increasing procedural complexity in therapeutic endoscopy. With the emergence of advanced techniques such as endoscopic ultrasound‐guided biliary drainage and pancreatic drainage, where procedure times can extend to 80 min or more,^12^, ^13^, ^14^, ^15^ the ability to maintain clear visualization while minimizing radiation exposure becomes crucial. The International Commission on Radiological Protection's ^16^, ^17^, ^18^ recommended annual dose limit of 20 mSv underscores the importance of such radiation‐reducing technologies.

This study has several limitations. First, the use of phantoms, while allowing for standardized comparisons, may not fully replicate the complexities of clinical practice. The experiments were conducted with optimal tube and detector alignment, which doesn't account for potential image quality degradation due to misalignment often encountered in practice. Such misalignments, caused by individual patient differences, can lead to image defocusing. To validate the utility of the CE protocol in actual ERCP procedures, prospective studies, including our ongoing randomized controlled trial, are necessary. These studies will help evaluate the protocol's performance across various clinical scenarios and patient populations. Secondly, the phantom's rotation speed of approximately 30 rpm likely exceeds the typical movement speed of devices within bile or pancreatic ducts. This discrepancy was unavoidable due to the fixed rotation speed of the phantom. While this higher speed provides a rigorous test of dynamic resolution, it may not precisely mirror clinical conditions. Third, this study did not evaluate contrast medium visibility, which is a crucial factor in clinical ERCP procedures. Future studies should incorporate contrast medium visibility assessment to provide a more comprehensive evaluation of the CE protocol's clinical utility. Fourth, this study evaluated only the CE protocol implemented in the Ultimax‐i system. As of December 2024, similar protocols that enhance contrast processing have been introduced in X‐ray systems from other manufacturers (e.g., WOW protocol by FUJIFILM and DeEP protocol by Shimadzu). However, since only the CE protocol was assessed in this study, the effectiveness of other protocols remains unclear. Future comparative studies of these various protocols could help establish optimal approaches for reducing radiation exposure while maintaining image quality. Fifth, this study did not evaluate the variability of the assessment results. While inter‐rater reliability metrics such as Fleiss's kappa would typically be calculated for multiple evaluators, the single‐evaluation design and small sample size of this study precluded meaningful reliability analysis.

## CONCLUSION

Our phantom‐based evaluation demonstrated that three CE protocols—LOW‐7.5 fps, MID‐3.75 fps, and LOW‐3.75 fps—achieve superior dynamic spatial resolution compared to the standard protocol while operating at lower radiation doses. However, prospective randomized controlled trials comparing both protocols in clinical ERCP procedures will be essential to validate their real‐world impact.

## CONFLICT OF INTEREST STATEMENT

Eisuke Iwasaki serves as an Editor of *DEN*. The authors declare a conflict of interest related to this article.

## ETHICS STATEMENT

Approval of the research protocol by an Institutional Reviewer Board: N/A.

## PATIENT CONSENT STATEMENT

N/A.

## CLINICAL TRIAL REGISTRATION

N/A.

## Supporting information



Evaluation of dynamic spatial resolution using a rotating wire phantom. The dose rate values (mGy/min) displayed on the screen during fluoroscopy are real‐time estimates that account for various factors including patient body size. These displayed values differ from the precise measurements obtained using the dosimeter (reported in Table 1), which were used for all comparative analyses in this study. For the dose measurements, we used the method shown in Figure 1.
